# Exploitation of a Type 1 Toxin–Antitoxin System as an Inducible Counter-Selective Marker for Genome Editing in the Acetogen *Eubacterium limosum*

**DOI:** 10.3390/microorganisms11051256

**Published:** 2023-05-10

**Authors:** James Millard, Alexander Agius, Ying Zhang, Philippe Soucaille, Nigel Peter Minton

**Affiliations:** 1 BBSRC/EPSRC Synthetic Biology Research Centre (SBRC), Biodiscovery Institute, School of Life Sciences, University of Nottingham, Nottingham NG7 2RD, UKmrzyz@exmail.nottingham.ac.uk (Y.Z.); soucaille@insa-toulouse.fr (P.S.); 2 Institut National des Sciences Appliquées, Toulouse Biotechnology Institute (TBI), Université de Toulouse, 31400 Toulouse, France

**Keywords:** *Eubacterium limosum*, *Eubacterium callanderi*, toxin–antitoxin systems, RelBE, CRISPR, gene editing

## Abstract

Targeted mutations in the anaerobic methylotroph *Eubacterium limosum* have previously been obtained using CRISPR-based mutagenesis methods. In this study, a RelB-family toxin from *Eubacterium callanderi* was placed under the control of an anhydrotetracycline-sensitive promoter, forming an inducible counter-selective system. This inducible system was coupled with a non-replicative integrating mutagenesis vector to create precise gene deletions in *Eubacterium limosum* B2. The genes targeted in this study were those encoding the histidine biosynthesis gene *hisI*, the methanol methyltransferase and corrinoid protein *mtaA* and *mtaC*, and *mtcB*, encoding an Mttb-family methyltransferase which has previously been shown to demethylate L-carnitine. A targeted deletion within *hisI* brought about the expected histidine auxotrophy, and deletions of *mtaA* and *mtaC* both abolished autotrophic growth on methanol. Deletion of *mtcB* was shown to abolish the growth of *E. limosum* on L-carnitine. After an initial selection step to isolate transformant colonies, only a single induction step was required to obtain mutant colonies for the desired targets. The combination of an inducible counter-selective marker and a non-replicating integrative plasmid allows for quick gene editing of *E. limosum*.

## 1. Introduction

*Eubacterium limosum* is a Gram-positive, non-spore-forming anaerobe which is able to utilise single-carbon substrates such as methanol and CO_2_, producing acetate and butyrate [1]. These substrates are assimilated via the Wood–Ljungdahl pathway (or reductive acetyl-CoA pathway) [2]. Noted for its mucoid phenotype, *E. limosum* was isolated from human stool samples and published originally as *Bacteroides limosus* [3]. Its ability to assimilate single-carbon substrates and its product profile make *E. limosum* of biotechnological significance. Methanol in particular is a substrate of interest because it does not suffer the same mass-transfer limitations as gaseous substrates [4]. In addition to the acids described above, *E. limosum* has also been reported to produce butanol when growing on a mixture of methanol and formate [5]. 

Tools for the genetic manipulation of *E. limosum* have been developed only relatively recently. These have included the use of various autonomous plasmids to demonstrate the utility of lactose- and anhydrotetracycline-induced promoters for the expression of the CreiLOV fluorescent reporter, the use of oxygen-independent fluorescent reporter genes to study gene expression as well as plasmid-based metabolic engineering strategies for the production of acetone and butanol [6]. The complete genome sequences (with accompanying transcriptomic data) for *E. limosum* strain ATCC 8486 (the type strain) and B2 have been published [7,8], but of particular importance is the ability to exploit this information through genome editing based on gene knock-out (KO) and knock-in (KI) strategies. Accordingly, allelic exchange protocols for the generation of such directed mutants have been developed that employed CRISPR-Cas9 [9]. In this protocol, however, cells carrying the mutant allele were selected on the basis of the acquisition of antibiotic resistance as a consequence of an *ermB* gene carried within the mutant allele. The generation of such insertional mutants, however, is far from ideal as the inserted gene can potentially alter the phenotype of the mutant as a consequence of polar effects on flanking genes. Accordingly, markerless deletion mutants are generally preferred. 

In the present study, we set out to develop an efficient KO system that would allow the isolation of markerless, in-frame deletion mutants following allelic exchange. Such clonal populations are classically selected using a counter-selection marker which in a number of cases has been derived from a toxin–antitoxin (TA) system. TA systems are ubiquitous in bacteria and consist of a toxin gene with a cognate labile antitoxin gene. They are divided into eight classes which are distinguished by the nature of their encoded toxins or antitoxins (whether protein or RNA-based) and by the mode of action of these components [10]. Plasmid-borne TA systems were first identified in 1983 [11], with chromosomally integrated TA systems being identified in the 1990s [12]. 

The exploitation of a TA system in KO strategies essentially involves the inducible production of the toxin component following double cross-over integration of the KO vector at the target site. Those cells that survive the lethal effects of the toxin represent those in which the integrated vector has excised and generated progeny carrying either the parental or mutant allele. The two types of cells are distinguished using an appropriate diagnostic PCR reaction. Examples of the use of a TA system in this mode include the use of the *Escherichia coli* MazF toxin to generate markerless gene knock-ins and in-frame deletions in *Bacillus subtilis* [13], *Clostridium acetobutylicum* [14] and *Clostridium pasteurianum* [15] as well as a phage-derived system to make mutants in *Clostridium difficile* [16]. 

In this study, a RelBE-family TA system from *E. callanderi* KIST612 was repurposed as an inducible counter-selective marker for use in *E. limosum* B2. The inducible toxicity of this system was demonstrated, and a set of non-replicating mutagenesis vectors were constructed. These vectors were used to create in-frame deletions of *hisI* (phosphoribosyl-AMP cyclohydrolase/phosphoribosyl-ATM pyrophosphatase), *mtaA* (a methyltransferase), *mtaC* (a corrinoid binding protein) and *mtcB* (encoding a recently described carnitine demethylase [17]). This mutagenesis method allows for the rapid modification of the *E. limosum* genome using easily assembled vectors. 

## 2. Materials and Methods

### 2.1. Bacterial Strains and Routine Culture of Bacteria

Unless specified otherwise, all references to *E. limosum* in this study refer to strain B2, (INSA, Toulouse, France). The use of B2 was expedient because it does not possess the mucoid phenotype which is characteristic of wild-type *E. limosum* ATCC 8486 [6]. All cultivation of *E. limosum* was performed in an anaerobic cabinet (Don Whitley Scientific, Bingley, UK). The growth medium used was derived from that of Pacaud and co-workers [18] and comprised NH_4_Cl (1.0 g/L), NaCl (1.5 g/L), L-cysteine hydrochloride (0.5 g/L), trace element solution (10 mL/L), macro-mineral solution (50 mL/L) and vitamin solution (10 mL/L). Glucose was added to a final concentration of 20 mM as a carbon source, except where otherwise noted. The trace element solution consisted of FeSO_4_·7H_2_O (0.1 g/L), MnCl_2_·4H_2_O (0.1 g/L), CaCl_2_·2H_2_O (0.17 g/L), CoCl_2_·2H_2_O (0.1 g/L), ZnCl_2_ (0.1 g/L), CuCl_2_ (0.02 g/L), H_3_BO_3_ (0.01 g/L), NaCl (1 g/L), Na_2_SeO_3_ (0.017 g/L), NiSO_4_·6H_2_O (0.026 g/L) and nitrilotriacetic acid (12.8 g/L). The macro-mineral solution consisted of KH_2_PO_4_ (6 g/L), NaCl_2_ (12 g/L), MgSO_4_·7H_2_O (2.4 g/L) and CaCl_2_·2H_2_O (1.6 g/L). The vitamin solution consisted of biotin (2 mg/L), lipoic acid (5 mg/L) and pantothenic acid (5 mg/L). The medium was supplemented with 1 g/L yeast extract except where otherwise noted. When growing with supplementary histidine, histidine was added to a final concentration of 2.5 mM. When growing on L-carnitine, the culture was additionally supplemented by casamino acids and sodium acetate as in the study of Kountz and co-workers [17]. Growth media were supplemented with thiamphenicol (Tm) when necessary, to a concentration of 15 μg/mL. To induce toxin activity and force plasmid loss, anhydrotetracycline (ATc) was supplemented to a final concentration of 100 ng/mL. All cultivation of *E. limosum* was conducted at 37 °C under anaerobic conditions in an anaerobic cabinet (Don Whitley Scientific Limited, UK). To assess the growth of *E. limosum* cultures, optical density readings at 600 nm were taken using either a Jenway 7305 UV–visible spectrophotometer (Cole-Parmer, Vernon Hills, IL, USA) or an Eppendorf BioSpectrometer (Eppendorf, Hamburg, Germany). Plasmid vectors, primers and oligonucleotides used in this study are listed in Table 1 and Table 2. Plasmids and their sequences may be sourced from www.plasmidvectors.com (accessed on 1 May 2023). 

### 2.2. High-Efficiency Electroporation of E. limosum

Competent cells of *E. limosum* were prepared by inoculating 500 mL of Pacaud medium (as described above) from an overnight preculture. The culture was grown to an OD600 of 0.2–0.5 and harvested by centrifugation. Centrifugation steps were carried out on the benchtop, but cells were returned to the anaerobic cabinet for resuspension steps. All centrifugation steps were carried out at 7000× *g* in a pre-chilled centrifuge at 4 °C. Cells were centrifuged and resuspended twice in ice-cold anaerobic electroporation buffer (EPB; 270 mM sucrose, 5 mM Na_2_HPO_4_/NaH_2_PO_4_ and pH 6.8). Following these wash steps, cell pellets were resuspended in 2 mL EPB with 10% *v*/*v* DMSO and stored in 100–300 μL aliquots at −80 °C for later use. To transform *E. limosum*, 100 μL aliquots of competent cells were thawed on ice under anaerobic conditions. Prepared plasmid DNA (~1 μg) was mixed with the thawed competent cells in a chilled electroporation cuvette with a 2 mm gap width, and the mixture was incubated on ice for five minutes. Electroporations were conducted using a Bio-Rad Gene Pulser Xcell (Bio-Rad Laboratories, Hercules, CA, USA) operating in time constant mode (voltage 1.7 kV, time constant 6 ms). Following electroporation, cells were recovered in 1 mL medium for 2–4 h prior to plating. Transformants were selected for with Tm at a concentration of 15 μg/mL. This transformation protocol permitted transformation efficiencies sufficient to employ non-replicative vectors in this study. Transformation of the shuttle vector pMTL83151 yielded an average of 2.09 × 10^5^ CFU/μg DNA (*n* = 3). When transforming non-replicating ‘suicide’ vectors such as pMTL-JM201, transformation efficiencies of approximately 1 × 10^2^ CFU/μg were observed.

### 2.3. Cloning of the Toxicity Assay Vector pMTL-JM101

All primers were designed using the NEB Assembler Tool (https://nebuilder.neb.com, accessed on 1 May 2023) with standard assembly parameters. Primers bb1_fwd/bb1_rev were used to amplify a linear fragment from pMTL83151 [19]. The sequences encoding the *E. callanderi* RelE toxin (ELI_RS06335, WP_013379749.1) and cognate RelB antitoxin (ELI_RS06330, WP_013379748.1) were obtained from the genome published by Roh and co-workers [20] (NCBI accession NC_014624). The antitoxin (with accompanying TA system promoter) and toxin were synthesised as separate fragments (IDT, Coralville, IA, USA). These toxin and antitoxin fragments were used as PCR templates and amplified with primer sets tox_fwd/tox_rev and atox_fwd/atox_rev, respectively. The tetracycline-inducible promoter system (Tet system) was based on that employed by Fagan and Fairweather in *Clostridioides difficile* [21]. Primers tet_fwd and tet_rev were used to amplify the Tet system from pMTL-tet3no (SBRC Nottingham). NEBuilder® HiFi DNA Assembly Master Mix (NEB, Ipswich, MA, USA) was used to assemble the DNA fragments containing the Tet system, toxin, antitoxin (with promoter) and the linearised pMTL83151 backbone to produce the complete pMTL-JM101 plasmid. The plasmid is available from www.plasmidvectors.com (accessed on 1 May 2023).

### 2.4. Cloning of the hisI Knockout Vector pMTL-JM201-hisI

Primers bb2_fwd/bb2_fwd were used to amplify a linear fragment from a dilution series of pMTL-JM101. This fragment contained the entirety of pMTL-JM101 except for the Gram-positive replicon and served as the backbone of the pMTL-JM201 knockout vectors. The amplified product from the PCR with the most dilute template was purified for use in the subsequent NEBuilder® HiFi assembly. Primer sets hisI_lha_fwd/rev and hisI_rha_fwd/rev were used to amplify the homology arm fragments. Homology arms were 1.0 kb in each case. The backbone fragment and homology arms were used in a three-part HiFi assembly to produce pMTL-JM201-*hisI*. The backbone of pMTL-JM201 retained the *catP* selective marker from pMTL83151, conferring resistance to thiamphenicol.

### 2.5. Cloning of the mtaA and mtaC Knockout Vectors pMTL-AA201-mtaA and pMTL-AA201-mtaC

Vectors pMTL-AA201-mtaA and pMTL-AA201-mtaC were constructed as described for pMTL-JM201-*hisI* above, except that primer sets mtaA_lha_fwd/rev, mtaA_rha_fwd/rev, mtaC_lha_fwd/rev and mtaC_rha_fwd/rev were used to amplify homology arm fragments (see Table 2, above) of approximately 0.75 kb in each case. Fragments were assembled using NEBuilder® as described above.

### 2.6. Cloning of the mtcB Knockout Vector pMTL-JM201-mtcB

Primers were designed as above. Primer sets *mtcB*_lha_fwd/*mtcB*_lha_rev and *mtcB*_rha_fwd/*mtcB*_rha_rev were used to amplify homology arms flanking the *E. limosum mtcB* gene (WP_038351887.1). Fragments were assembled using NEBuilder® as described above. Homology arms were 1.0 kb in length, with 3 bp overlaps with *mtcB*, resulting in a 6 bp scar containing the start and stop codons in the mutant chromosome after a double recombination event.

### 2.7. In Vivo RNA Staining with Thioflavin T

A method for staining bacterial RNA in vivo was employed based on that published by Sugimoto et al. [22]. *E. limosum* cells were harvested with centrifugation (12,000× *g*, 1:00 min) and resuspended in 25 μM thioflavin T in phosphate-buffered saline (PBS). The resuspended cells were incubated at room temperature for 5:00 min. Stained cells were washed twice in PBS and 200 μL per sample transferred to a 96-well plate. The plates were read using a Tecan Infinite® M1000 plate reader (Tecan AG, Männedorf, Switzerland). An excitation wavelength of 438 nm was used. Fluorescence intensities were recorded at 490 ± 5 nm. Optical density readings were collected at 600 nm for each well and used to normalise the fluorescence readings. 

## 3. Results

### 3.1. Demonstration of E. callanderi RelE toxicity in E. limosum

**Figure 1:**
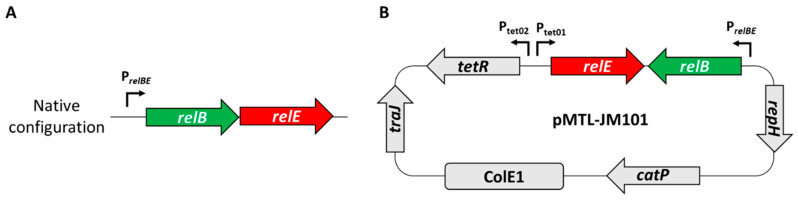
(**A**) The RelBE type 1 toxin–antitoxin (TA) system from *E. callanderi* KIST612. (**B**) The toxicity assay plasmid pMTL-JM101, with the expression of *relE* driven by an anhydrotetracycline (ATc)-inducible promoter system. Expression of *tetR* and *relE* is via Ptet01/02 which are inhibited by TetR until ATc is added. Expression of *relB* remains under the control of the native promoter of the TA system.

**Figure 2:**
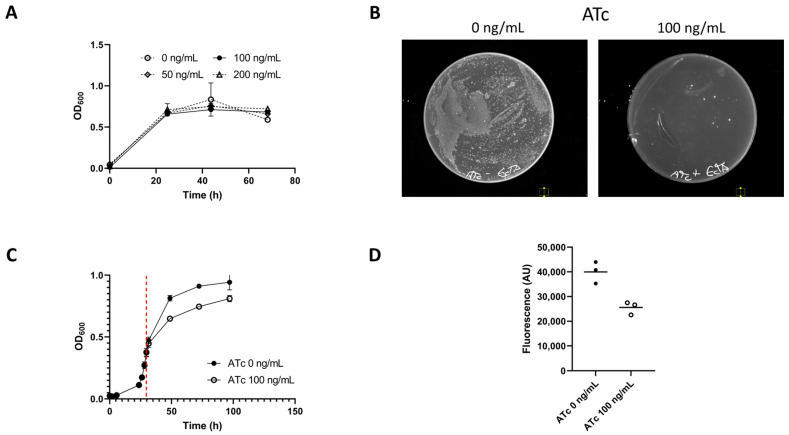
(**A**) Growth of *E. limosum* B2 on glucose (20 mM) in a medium supplemented with varying concentrations of anhydrotetracycline (ATc). Points represent the mean average (*n* = 3). Error bars represent the standard deviation. (**B**) *E. limosum* pMTL-JM101 growth on solid medium ± ATc. (**C**) Growth of *E. limosum* pMTL-JM101 in the presence and absence of ATc. Cultures were grown to the mid-exponential phase and then divided in half. One-half of each replicate was spiked with ATc to a final concentration of 100 ng/mL (indicated by the red line). Data points represent mean average values (*n* = 3). Error bars represent the standard deviation. (**D**) Fluorescence at 490 nm of cells harvested from (**C**) at 96 h and stained with thioflavin T. Bars represent the mean average of measurements (*n* = 3). Fluorescence measurements were normalised based on the sample optical density at 600 nm.

The toxin assay vector pMTL-JM101 (Figure 1) was transformed into *E. limosum* B2 and the effect of toxin induction was tested on both liquid and solid medium (Figure 2). When plated to a selective medium (15 μg/mL Tm) ± ATc, *E. limosum* pMTL-JM101 showed greatly reduced colony formation when toxin expression was induced (Figure 2). When ATc was added to the growing cultures of *E. limosum* pMTL-JM101 a growth impact was observed, though OD was observed to increase. This is consistent with other studies in which both toxin and antitoxin are expressed heterologously [23]. When cells were harvested from these cultures and stained with thioflavin T to reveal their RNA content [22], the fluorescence of the harvested cells at 490 nm was reduced by an average of 1.6-fold in cells in which the toxin had been induced. This supports the prediction that the *E. callanderi* RelE is an endoribonuclease. A previous study by Shin and co-workers observed no growth inhibition by ATc at concentrations of 30 ng/mL [9]. We additionally observed no inhibition of the growth of wild-type *E. limosum* B2 at ATc concentrations of up to 200 ng/mL (Figure 2). Observed impacts on colony formation or liquid growth can therefore be attributed to the induction of the plasmid-borne toxin gene. 

### 3.2. Exemplification of relE as a Counter-Selection Marker

Having both demonstrated the toxicity of RelE to *E. limosum* B2 and that its toxic effects could be induced by an ATc-regulated promoter, we tested its utility as a counter-selection marker in the isolation of markerless in-frame deletion mutants. For our proof-of-principle studies, we selected a target gene that should give a clear and easily detected auxotrophic phenotype. Accordingly, we targeted the gene (*hisI*, WP_013378694.1) encoding a homologue of phosphoribosyl-AMP cyclohydrolase/phosphoribosyl-ATM pyrophosphatase (HisI), a bifunctional enzyme which catalyses the second and third steps in histidine biosynthesis. Its inactivation would be predicted to result in cells that needed exogenous histidine in the growth medium.

**Figure 3:**
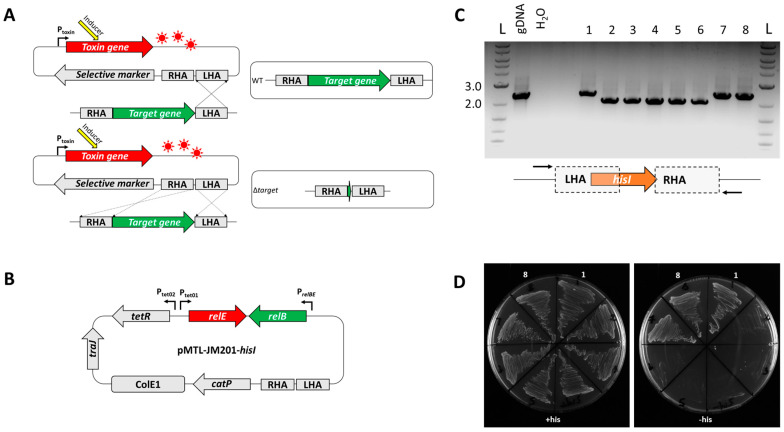
Isolation and characterisation of *E. limosum* B2 Δ*hisI* mutants. (**A**) Scheme for mutant generation. Following the integration of the non-replicating knockout vector, toxin induction will prevent cell growth on a non-selective medium unless the integrated plasmid is excised. This can occur due to (i) a recombination event at the original locus of recombination (resulting in a wild-type revertant) or (ii) recombination at the adjacent homology arm (resulting in the deletion of the region between the homology arms. (**B**) The *hisI* knockout vector pMTL-JM201-*hisI*. (**C**) Screening of hisI deletion mutants. Mutants were screened using primers *hisI*_ext_fwd and *hisI*_ext_rev. The ladder is NEB Quick-Load® Purple 1 kb Plus. (**D**) Plates showing growth of *E. limosum* B2 ATcR colonies on defined medium in the presence (+) and absence (−) of histidine. Plate sectors 1–8 correspond to the sample lanes in the gel image above.

The required plasmid to KO *hisI* (pMTL-JM201-*hisI*) was constructed (Materials and Methods) and transformed into *E. limosum.* The plasmid featured symmetrical 1.0 kb homology arms to allow integration into the *E. limosum* chromosome. Integrant colonies were selected on the basis of thiamphenicol resistance (Tm^R^) on Pacaud agar medium (as described above) supplemented with thiamphenicol (15 μg/mL) and histidine (2.5 mM). Tm resistance was conferred by the presence of the selective marker *catP* on the plasmid backbone. Colonies were then restreaked onto fresh medium lacking Tm but containing ATc (100 ng/mL). A total of eight colonies were screened both by patch plating onto Pacaud agar medium with and without histidine supplementation and by PCR screening with primer pair *hisI*-ext-fwd/rev (see Table 2, above, and Figure 3, below). The PCR bands generated indicated that, as expected, they comprised a mixture of mutant and restored wild-type clones. DNA from five of the eight clones produced a 2.3 kb DNA fragment when screened and did not require histidine supplementation for effective growth on the Pacaud medium. The remaining three colonies generated a smaller 2.0 kb DNA fragment in the PCR screen, indicative of the desired 309 bp in-frame deletion within *hisI*. These deletion mutants required exogenous histidine in the medium for growth (Figure 3). Sanger sequencing of the amplicon confirmed the presence of the expected in-frame deletion, and the mutant was designated *E. limosum* B2 Δ*hisI*. 

### 3.3. Deletion of mtaA and mtaC Genes

Having established proof of principle by targeting *hisI* we chose two further genes which on the basis of encoded homologies are likely to be essential for growth on methanol, and in particular methyltransferases and their associated corrinoid proteins. These allow acetogens to incorporate methyl groups from various substrates into the Wood–Ljungdahl Pathway (WLP). A methanol methyltransferase was previously described in *E. callanderi* [24] and more recently in *A. woodii* [25]. Accordingly, we targeted *mtaA* (WP_038352459.1) and *mtaC* (WP_038352387.1) which respectively encode methylcobalamin:tetrahydrofolate methyltransferase (MtaA) and the associated corrinoid-binding protein (MtaC) which transfers methyl groups to tetrahydrofolate. 

**Figure 4:**
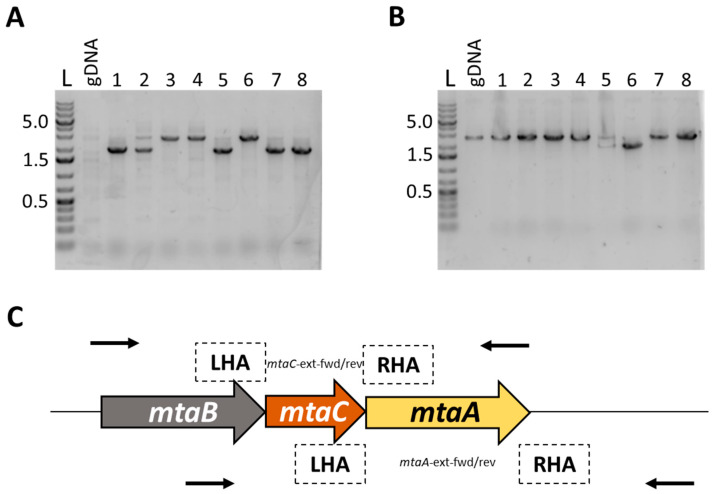
Screening of (**A**) *mtaA* and (**B**) *mtaC* deletion mutants. ATc^R^ colonies were screened using primer pairs *mtaA*-ext-fwd/rev and *mtaC*-ext-fwd/rev, respectively. Ladder: Generuler 1 kb Plus (Thermo Fisher, Waltham, MA, USA). Sample lanes are indicated using brackets. (**C**) Schematic representation of the *mta* operon, showing primer binding sites and homology arm locations. LHA/RHA: left/right homology arms. Refer to Table 2 for primer nucleotide sequences.

TA-based KO plasmids targeting each gene were made (Methods) in the vector pMTL-JM201 and the two resultant plasmids pMTL-AA201-*mtaA* and pMTL-AA201-*mtaC* were transformed into *E. limosum*. These plasmids featured shorter homology arms than pMTL-JM201-*hisI*, 753/750 bp and 762/760 bp in length, respectively. Isolated Tm^R^ transformants were streaked onto plates containing ATc and eight ATc^R^ clones were screened for the presence of the desired KO by PCR using primer pairs mt2-ext-fwd/rev and cop-ext-fwd/rev, respectively (see Table 2, above, and Figure 4, below). In the case of *mtaC*, the PCR screen revealed that six of the eight clones tested were mutants on the basis of the 2.0 kb fragment generated compared to the larger wild-type band of 2.6 kb. By contrast, of the eight ATc^R^ clones transformed with pMTL-AA201-*mtaC*, just two were mutants, with the remaining six being wild-type. The identity of the designated mutants was confirmed by Sanger sequencing of the PCR-amplified DNA band and a random representative of each type was selected and designated *E. limosum* B2 Δ*mtaC* and Δ*mtaA*, respectively. 

**Figure 5:**
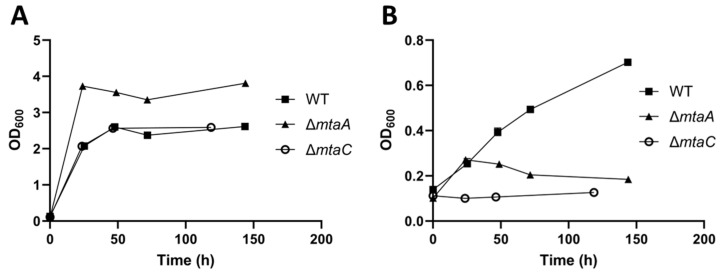
Growth of wild-type *E. limosum* and deletion mutants Δ*mtaA* and Δ*mtaC* on (**A**) 60 mM glucose and (**B**) 200 mM methanol. Points represent mean average OD600 readings (*n* = 3). Error bars represent the standard deviation.

The two isolated mutants were grown with either glucose or methanol as their carbon source to determine whether the deletion of *mtaA* or *mtaC* had an impact on their ability to grow autotrophically (Figure 5). No deleterious effect on glucose growth was observed for either deletion mutant. By contrast, no growth was observed for both mutants when growing on 200 mM methanol, confirming their hypothesised essential role in the assimilation of this C1 feedstock. 

### 3.4. Deletion of Carnitine Demethylase Gene mtcB

As a final test of the system’s utility in *E. limosum*, the gene *mtcB* encoding an MttB-family carnitine demethylase (WP_038351887.1) was selected as a target. A 2020 study found that this gene was upregulated when *E. limosum* ATCC 8486 was grown with carnitine as the growth substrate and that MtcB was able to demethylate L-carnitine in vitro [17].

**Figure 6:**
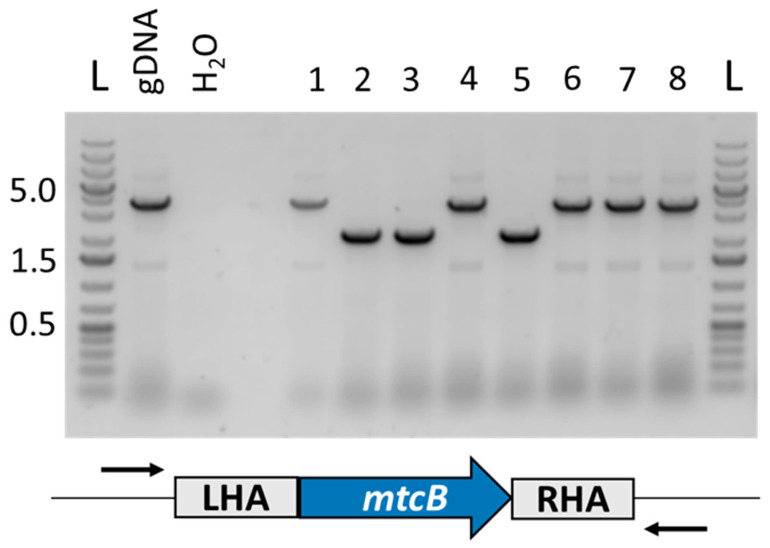
Diagnostic PCR of eight ATcR colonies using primers *mtcB*_ext_fwd and *mtcB*_ext_rev. Samples 2, 3 and 5 show band sizes consistent with the deletion of *mtcB* (wild-type 3637 bp; deletion 2182 bp). L: Generuler 1 kb Plus (Thermo Fisher).

The knockout vector pMTL-JM201-*mtcB* was transformed into *E. limosum* B2 and Tm^R^ colonies were obtained. Two independent transformations were conducted. Transformant colonies were restreaked to a non-selective medium supplemented with ATc (100 ng/mL), as above. The resulting colonies were screened with primers with binding sites adjacent to the locus of recombination. A representative gel from one such transformation is shown in Figure 6, on which three of the eight clones screened are mutants, an assumption confirmed by Sanger sequencing of the 3637 bp PCR band which showed the desired 1455 bp deletion.

**Figure 7:**
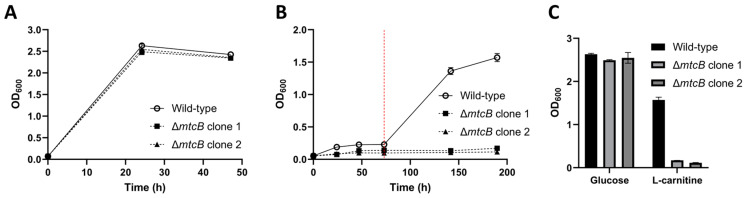
Phenotypic characterisation of *E. limosum* Δ*mtcB*. Two biologically independent Δ*mtcB* mutants are shown. (**A**) Growth of *E. limosum* wild-type and Δ*mtcB* using glucose (60 mM) as substrate. (**B**) Growth of *E. limosum* wild-type and Δ*mtcB* using L-carnitine (50 mM) as a substrate. The red line indicates the spiking of all cultures with an additional 50 mM L-carnitine at 73 h. (**C**) Maximum OD600 values attained in (**A**,**B**). All measurements shown are mean average (*n* = 3). Error bars represent the standard deviation.

The relative growth phenotype, compared to the wild-type, of two independent Δ*mtcB* mutants was investigated using either glucose (60 mM) or L-carnitine (50 mM) as the carbon source (Figure 7). Cultures growing on L-carnitine received a spike of additional L-carnitine at 73 h. All cultures were inoculated from precultures grown on glucose. Growth on glucose was not affected by the deletion of *mtcB*. By contrast, it was found that growth with L-carnitine as substrate was significantly impacted in the Δ*mtcB* mutants, with these cultures reaching peak OD_600_ values of 0.170 and 0.114, respectively, after 214 h, relative to 1.571 for the wild-type. This is consistent with the previously reported role of MtcB as a carnitine demethylase [17]. 

## 4. Discussion

In the present study, we have devised a simple and rapid method for the isolation of markerless gene deletions in *E. limosum* by an allelic exchange that exploits an inducible toxin–antitoxin system as a heterologous counter-selectable marker. This method is enabled through the use of suicide vectors, a consequence of a highly efficient transformation protocol allowing for 10^5^ transformant clones per μg DNA when transforming replicating plasmids. When transforming non-replicative ‘suicide’ plasmids, approximately 10^2^ transformants per μg DNA were obtained. This allows the direct selection of single crossover integrants. Cells in which a second recombination event has occurred, leading to the excision of the non-replicating plasmid and its subsequent loss, may be selected based on their resistance to ATc. The presence of ATc leads to the production of the RelB-family toxin, allowing only those cells that lose the integrated plasmid and toxin gene to survive. As in all such equivalent systems, the integrated plasmid excises as a consequence of recombination between one of the two sets of homology arms. The involvement of one pair of homology arms leads to the replacement of the wild-type allele with the mutant allele from the original KO plasmid whereas excision involving the other pair of homology arms (those involved in the original plasmid integration event) generates the original KO plasmid and the wild-type chromosomal allele remains intact. The two types of plasmid-free populations are distinguished by PCR. All things being equal, one would expect a mutant:wild-type ratio of 50:50, or 4:4 if eight ATcR colonies are screened. Here, the mutant:wild-type ratio varied between 5:3 (*hisI*), 5:3 (*mtaA*), 2:6 (*mtaC*) and 3:5 (*mtcB*). In every case, screening just eight colonies was sufficient to isolate mutants. 

Having established the initial proof of principle by successfully performing an in-frame deletion within the *hisI* gene, attention was switched to genes likely involved in methanol assimilation, through the in-frame deletion of *mtaA* (WP_038352459.1) and *mtaC* (WP_038352387.1). Our data clearly showed that the products of both genes, methylcobalamin:tetrahydrofolate methyltransferase (MtaA) and the associated corrinoid-binding protein (MtaC) which transfer methyl groups to tetrahydrofolate, were required for growth on methanol as the sole carbon source. Additionally, the system was used to knock out the carnitine demethylase gene *mtcB* of *E. limosum*. MtcB was previously identified as the enzyme responsible for the demethylation of L-carnitine, with the transfer of the resulting methyl group into the Wood–Ljungdahl pathway occurring via its cognate corrinoid binding protein MtqC and methylcorrinoid:tetrahydrofolate methyltransferase MtqA [17]. This identification was supported in another previous study by proteomic evidence showing increased expression of MtcB during growth on L-carnitine, as well as carnitine-dependent methylation of MtqC by MtcB in vitro [26]. In this study, gene deletions were performed in biological triplicate and in each case the mutant strain was found to be an L-carnitine auxotroph. This result concurs with those of Kountz and co-workers, who identified MtcB as a carnitine demethylase [17]. A subsequent study from the same group indicated that *E. limosum* possesses at least one additional methyltransferase capable of demethylating L-carnitine, but that it is not expressed in response to L-carnitine supplementation [26]. At least 42 MttB superfamily methyltransferases are encoded by the genome of *E. limosum* ATCC 8486 [26]. 

The knockout method detailed in this study could provide a means of creating strains that are auxotrophic for the substrates associated with these methyltransferases. Using auxotrophic strains as a background, restoration of prototrophy could be coupled to the insertion of genes of interest using an allele-coupled exchange approach [27]. This suggests a strategy in which every methyltransferase gene whose encoded product has an identified substrate becomes a locus for the insertion of genes of interest. Extensive metabolic engineering of *E. limosum* could potentially be conducted in this way. 

The *E. callanderi* TA system used in this study follows the ‘canonical’ arrangement for Type II TA systems, in which the antitoxin gene precedes the toxin gene and both are transcribed bicistronically [28]. While ectopic overexpression of the toxin is lethal to *E. limosum*, it is not possible to draw specific conclusions about the role of this TA system in its native context in *E. callanderi*. It may serve as an anti-addiction module by neutralising compatible RelE toxins expressed from incoming plasmids and thereby preventing colonisation by those plasmids [29]. The observed effectiveness of the *E. callanderi* RelE toxin suggests that the TA system in its canonical configuration could be used to stabilise plasmids in *E. limosum* without the need for antibiotic selection. It was not possible to clone a toxin-only expression vector with the *E. callanderi relE* gene downstream of the Tet promoter and the *relB* antitoxin absent. This may be attributable to the leakiness of the Tet system in *E. limosum*. Previous studies have successfully cloned *relE* genes downstream of more stringent promoters, such as pBAD [23,30]. 

In conclusion, we have developed a simple and rapid genome editing system for making markerless, precise deletion mutants of *E. limosum* that overcomes the drawback of CRISPR-based systems described to date which require the inclusion of an antibiotic resistance gene within the mutant allele.

## Figures and Tables

**Table 1:** Plasmid vectors used in this study.

Vector	Purpose	Source
pMTL-JM101	Inducible toxin test vector used to demonstrate the efficacy of the *E. callanderi* toxin.	This study.
pMTL-JM201-*hisI*	TA knockout vector targeted against the histidine biosynthesis gene *hisI*.	This study.
pMTL-AA201-*mtaA*	TA knockout vector targeted against *mtaA*, a putative corrinoid:methyl-THF methyltransferase.	This study.
pMTL-AA201-*mtaC*	TA knockout vector targeted against *mtaC*, a corrinoid-binding protein associated with *mtaA*/B.	This study.
pMTL-JM201-*mtcB*	TA knockout vector targeted against *mtcB*, encoding a carnitine:corrinoid methyltransferase (WP_038351887.1).	This study.
pMTL83151	*E. coli*-*Clostridium* shuttle vector.	Heap et al. [19].
pMTL-tet3no	*E. coli*-*Clostridium* shuttle vector with tetracycline-inducible divergent promoter system.	SBRC Nottingham

**Table 2:** Primers and oligonucleotides used in this study.

Primer	5′-3′ Oligonucleotide Sequence	Function
bb1_fwd	gtacccggggatcctctag	Amplification of pMTL-JM101 backbone from pMTL83151
bb1_rev	cgagctcgaattcgtaatcatg	
tox_fwd	ggaaatacatatgaaaagctatgaggtg	Amplification of *E. callanderi relE*
tox_rev	tggtgaatgatcaattccatatatcttccag	
atox_fwd	atggaattgatcattcaccatatttctcctgc	Amplification of *E. callanderi relB*
atox_rev	tctagaggatccccgggtacgcaggaggcgatttgattttg	
tet_fwd	tgattacgaattcgagctcgttaagacccactttcacatttaag	Amplification of Tetracycline-inducible promoter system from pMTL-tet3no
tet_rev	agcttttcatatgtatttcctcctcttcaatatatttaag	
bb2_fwd	ggccggccagtgggcaag	Amplification of pMTL-JM201 vector backbone from pMTL-JM101.
bb2_rev	ttgtcaattgttcaaaaaaataatggcggcgcg	
*hisI*_lha_fwd	tttttttgaacaattgacaatggcggacccggtagagc	Primers for left homology arm of *hisI* deletion construct.
*hisI*_lha_rev	ataaggcttaattacggtagaagcaggaagtttcgc	
*hisI*_rha_fwd	ctaccgtaattaagccttattaaaaaaagaacc	Primers for right homology arm of *hisI* deletion construct.
*hisI*_rha_rev	aacttgcccactggccggccaatatcttttttggataaaatttgtg	
*mtaA*_lha_fwd	ttagtacacactgcgcgc	Primers for left homology arm of *mtaA* deletion construct
*mtaA*_lha_rev	aatgaaatcatcgggataggatgcctcctgttcgtc	
*mtaA*_rha_fwd	gacgaacaggaggcatcctatcccgatgatttcattctccaagt	Primers for right homology arm of *mtaA* deletion construct
*mtaA*_rha_rev	aactctccctccagctgttc	
*mtaA*_ext_fwd	aggaaatcaatgacgaagccct	External screening primers for confirming *mtaA* knockout.
*mtaA*_ext_rev	taagagattgatacgctccgcc	
*mtaA*_int	ataccatcaaggttatcaacgacgcagg	Internal sequencing primer for sequencing *mtaA* knockout locus.
*mtaC*_lha_fwd	caaggactgcgcctatgaagg	Primers for left homology arm of *mtaC* deletion construct
*mtaC*_lha_rev	ttcgtcaaaattttattcgctgtctgttttatatcctccgatttttgtttttattcct	
*mtaC*_rha_fwd	aaaacaaaaatcggaggatataaaacagacagcgaataaaattttgacgaa	Primers for right homology arm of *mtaC* deletion construct
*mtaC*_rha_rev	tcattttgccattggtgggg	
*mtaC*_ext_fwd	agactggggaatactttgcagg	External screening primers for confirming *mtaC* knockout.
*mtaC*_ext_rev	agttgacgtcgatataggtcgc	
*mtaC*_int	ggacatggacaaatggcatcctgaag	Internal sequencing primer for sequencing *mtaC* knockout locus.
*mtcB*_lha_fwd	tttttttgaacaattgacaatgctggcgcctgtaaagg	Primers for left homology arm of *mtcB* in-frame deletion.
*mtcB*_lha_rev	attgagcttacatttgtctctctccttaaatatctcaaaatcttttc	
*mtcB*_rha_fwd	gagacaaatgtaagctcaatggggatcg	Primers for right homology arm of *mtcB* in-frame deletion.
*mtcB*_rha_rev	aacttgcccactggccggcctttcggcaggatcaggaaag	
*mtcB*_ext_fwd	catccaaaaacaatgtgccgttgttgg	External sequencing primers for confirming *mtcB* knockout.
*mtcB*_ext_rev	gcgccgaatatatgaatgggcacc	
*mtcB*_int	ccagataagcgctgtattcaggatcatgg	Internal sequencing primer for sequencing *mtcB* knockout locus.

## Data Availability

Plasmids and their sequences are available at www.plasmidvectors.com (accessed on 1 May 2023).
